# Supplementary material for: Ferulic Acid Induces Keratin 6α via Inhibition of Nuclear β-Catenin Accumulation and Activation of Nrf2 in Wound-Induced Inflammation
Source: Biomedicines. 2021 Apr 22;9(5):459. doi: 10.3390/biomedicines9050459 (PMC8146113; doi:10.3390/biomedicines9050459)

Tables.

Table 1. Up-regulation of genes associated with wound induced keratins.

Probe name	Description	Gene symbol	Fold change
**Keratins**			
1422784_at	keratin 6A	*Krt6a*	14.6901
1423227_at	keratin 17	*Krt17*	7.9747
1424096_at	keratin 5	*Krt5*	7.3153
1423935_x_at	keratin 14	*Krt14*	8.1810

Fold change: BSL/SH normalized intensity ratio.

Table 2. Alteration of genes associated with extracellular matrix.

Probe name	Description	Gene symbol	Fold change
**Collagen**			
1455494_at	collagen, type I, alpha 1	*Col1a1*	2.5030
1423110_at	collagen, type I, alpha 2	*Col1a2*	2.6747
1427884_at	collagen, type III, alpha 1	*Col3a1*	3.4892
1418799_a_at	collagen, type XVII, alpha 1	*Col17a1*	2.4751
**Tight junction**			
1437932_a_at	claudin 1	*Cldn1*	2.1909
1434651_a_at	claudin 3	*Cldn3*	2.0510
**Adherens junction**			
1448261_at	cadherin 1	*Cdh1*	2.2079
**Desmosome**			
1435494_s_at	desmoplakin	*Dsp*	4.6570
1434534_at	desmocollin 3	*Dsc3*	2.1909
**Gap junction**			
1415801_at	gap junction protein, alpha 1	*Gja1*	-0.39884

Fold change: BSL/SH normalized intensity ratio.

Table 3. Alteration of genes associated with migration.

Probe name	Description	Gene symbol	Fold change
**Oncogene**			
1423240_at	Rous sarcoma oncogene	*Src*	2.1337
**Integrin linked kinases**			
1449942_a_at	integrin linked kinase	*Ilk*	2.0405
**Chemokine ligand**			
1417574_at	chemokine (C-X-C motif) ligand 12	*Cxcl12*	3.078
**Fibroblast growth factor**			

1422916_at	fibroblast growth factor 21	*Fgf21*	2.0611
**A disintegrin and metallopeptidase**			
1450105_at	a disintegrin and metallopeptidase domain 10	*Adam10*	-0.2529
1421857_at	a disintegrin and metallopeptidase domain 17	*Adam17*	-0.1847
**Wnt signaling**			
1448818_at	wingless-related MMTV integration site 5A	*Wnt5a*	-0.4180
1430533_a_at	catenin (cadherin associated protein), beta 1	*Ctnnb1*	-0.0786

Fold change: BSL/SH normalized intensity ratio.

Supplementary figures.

Supplementary Fig. 1

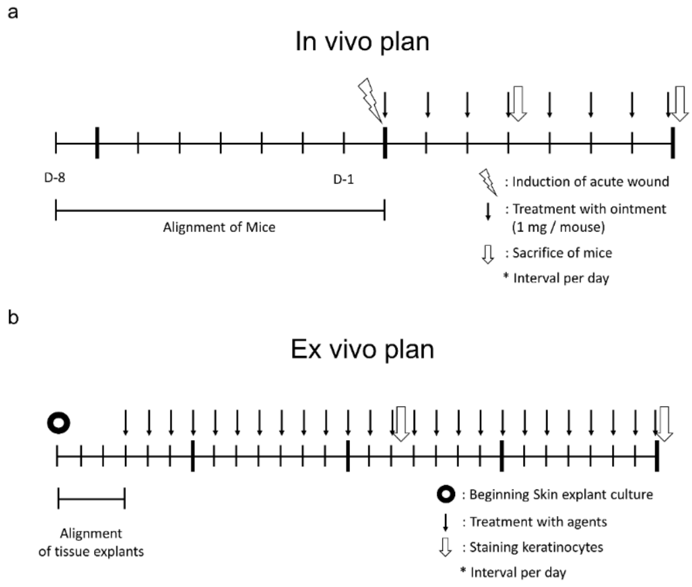

Supplementary Fig. 1. Plan of *in vivo* and *ex vivo*. (a) The schematic timeline of *in vivo* work was exhibited. During 7 days, we observed wound closure of mice back skin after post-wounding. (b) The schematic timeline of *ex vivo* work was represented. During 24 days, we observed and cultured skin explants, derived from mice back skin (P0). After 12 day or 24 days, we stained migrated cells, derived from skin explants using H&E solution.

Supplementary Fig. 2

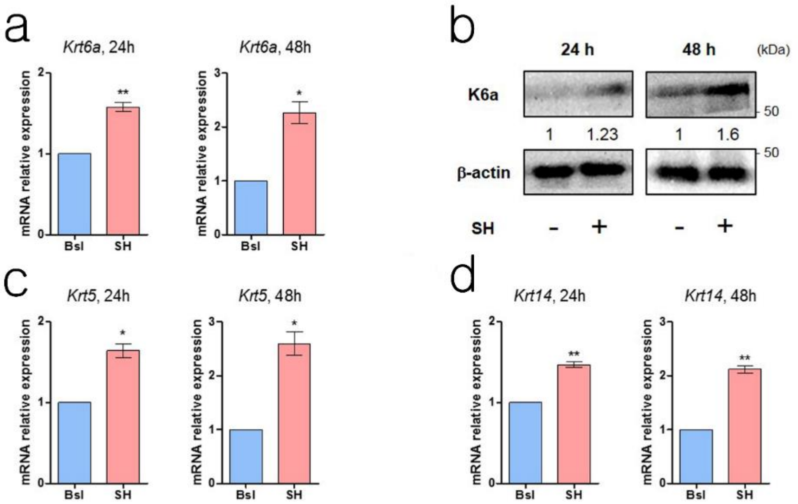

Supplementary Fig. 2. Transcriptomic analysis of keratins in primary keratinocytes. (a) In primary keratinocytes, mRNA of *Krt6a* was analyzed after treatment with SH. (b) Western blotting of Keratin 6 was induced after treatment with SH. (c) In primary keratinocytes, mRNA of *Krt5* was analyzed after treatment with SH. (d) In primary keratinocytes, mRNA of *Krt14* was analyzed after treatment with SH.

Supplementary Fig. 3

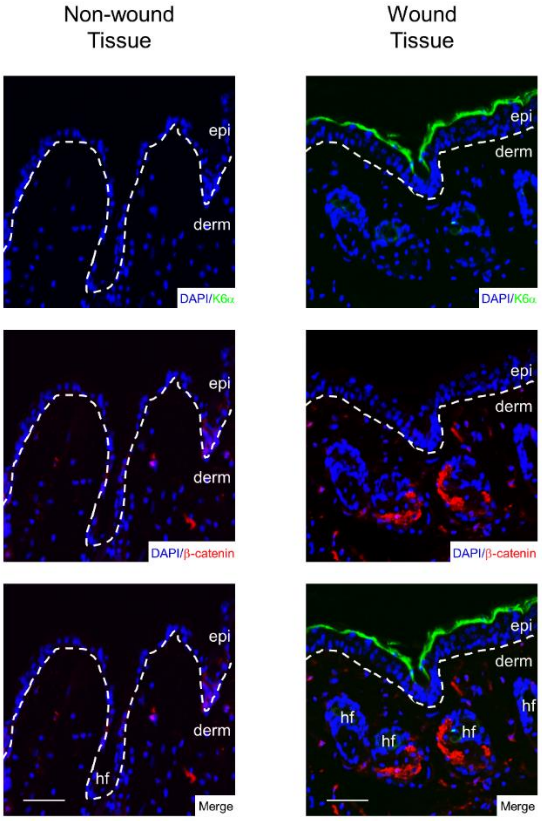

Supplementary Fig. 3. Immunofluorescence staining between non-wound and wound tissue.Between non-wound and wound tissue, we performed immunofluorescence staining of wound-induced keratin, K6α (wound marker, green) and β-catenin (red), associated with proliferation of fibroblasts on wound edge.

Supplementary Fig. 4

a

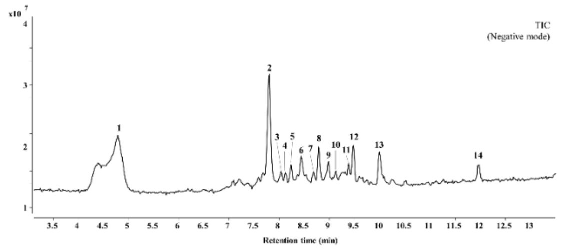

b

No.	*m/z*	RT	Formula	Score
1	178.0515	4.856	C9H9NO3	98.11
2	242.141	7.789	C13H17N5	96.77
3	345.1557	8.037	C16H26O8	99.11
4	345.1559	8.12	C16H26O8	98.1
5	413.2185	8.236	C21H34O8	98.27
6	345.1559	8.451	C16H26O8	99.12
7	351.2179	8.683	C20H32O5	98.15
8	473.1456	8.783	C24H26O10	99.46
9	325.1298	8.965	C16H22O7	97.15
10	327.1455	9.114	C16H24O7	98.92
11	329.1609	9.379	C16H26O7	99.41
12	220.0985	9.479	C12H15NO3	99.16
13	297.1135	10.009	C18H18O4	98.77
14	194.0833	11.964	C10H13NO3	94.01

c

No.	*m/z*	RT	Area	Height
2	242.141	7.789	102816081.93	18440626.31
2-1	193.0506	7.938	281128.48	81731.73

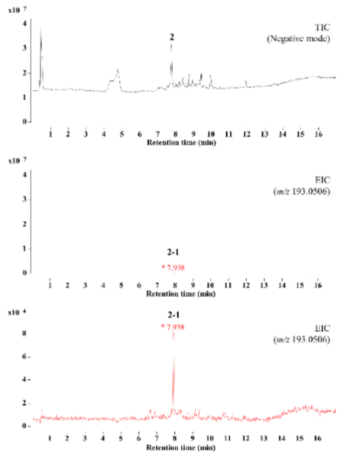

Supplementary Fig. 4. Total ion chromatogram of *shilajit* (a) Total ion chromatogram of *shilajit* extract by LC/MS analysis and chemicals. (b) The represented constituents were detected by *shilajit*. (c) The extracted chromatogram on 193. 0506.

Western blotting on Figure . 3

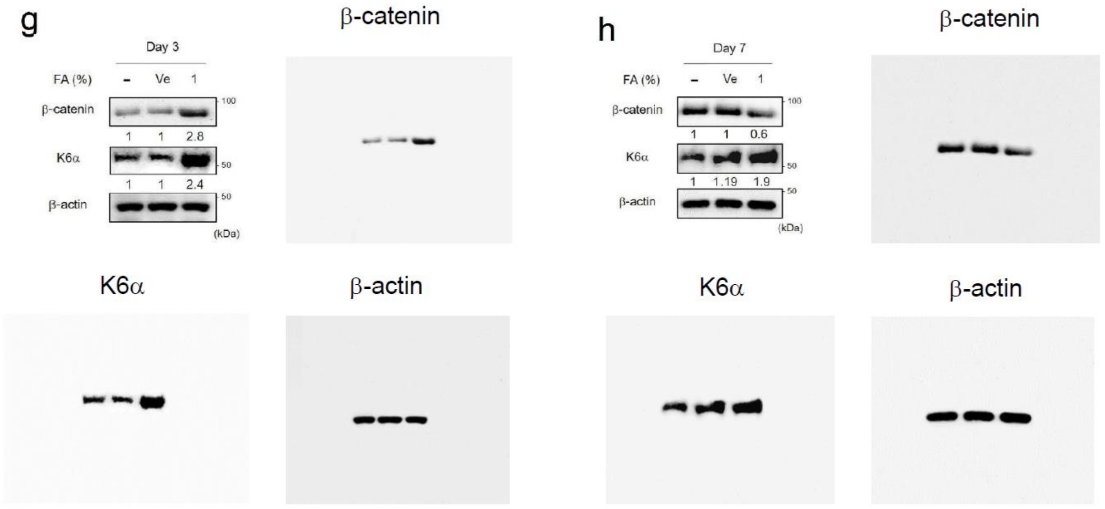

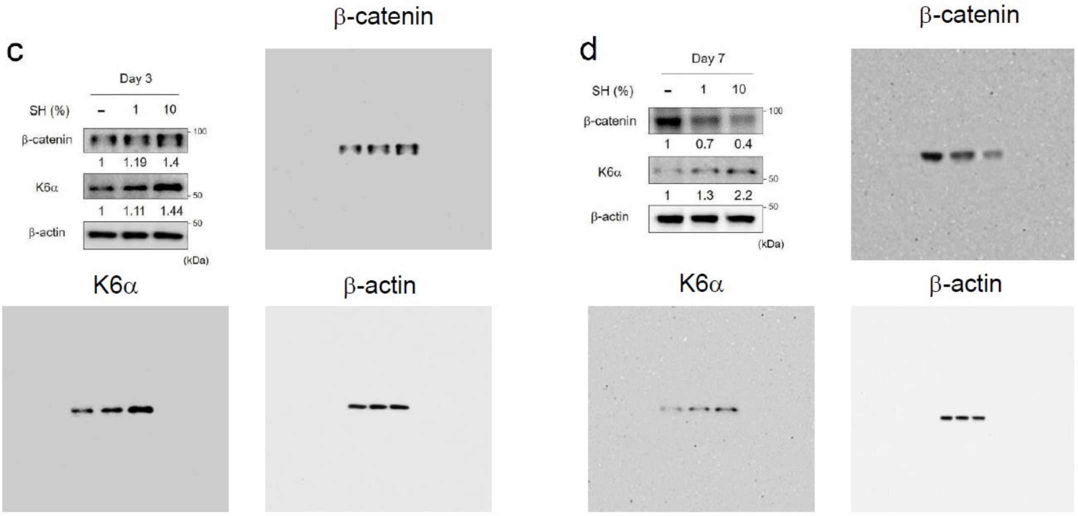

Western blotting on Figure . 4

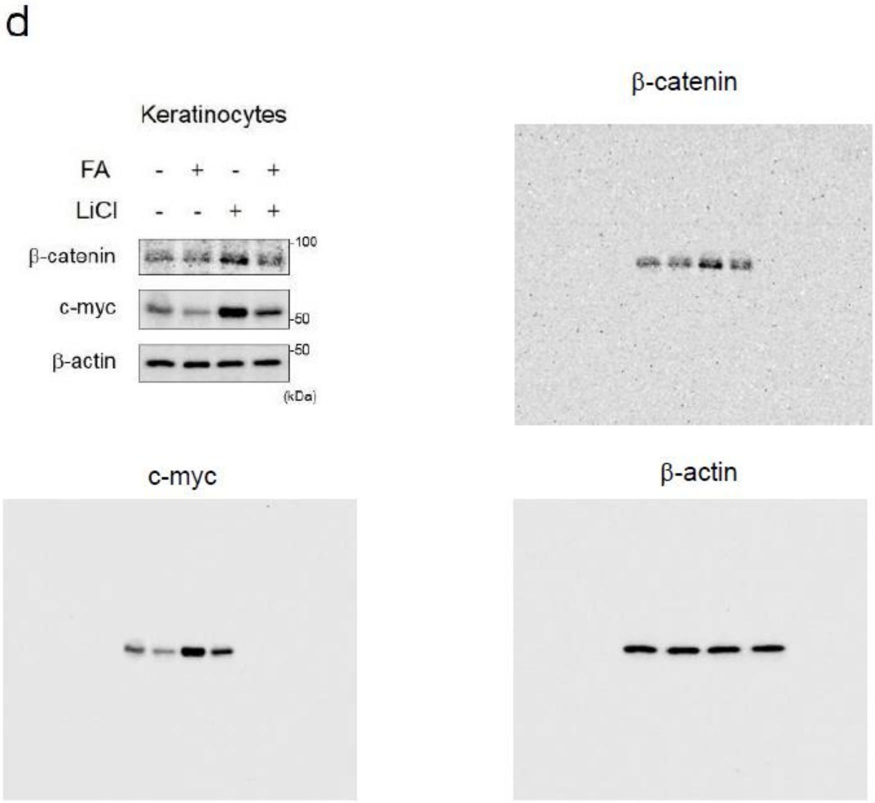

j

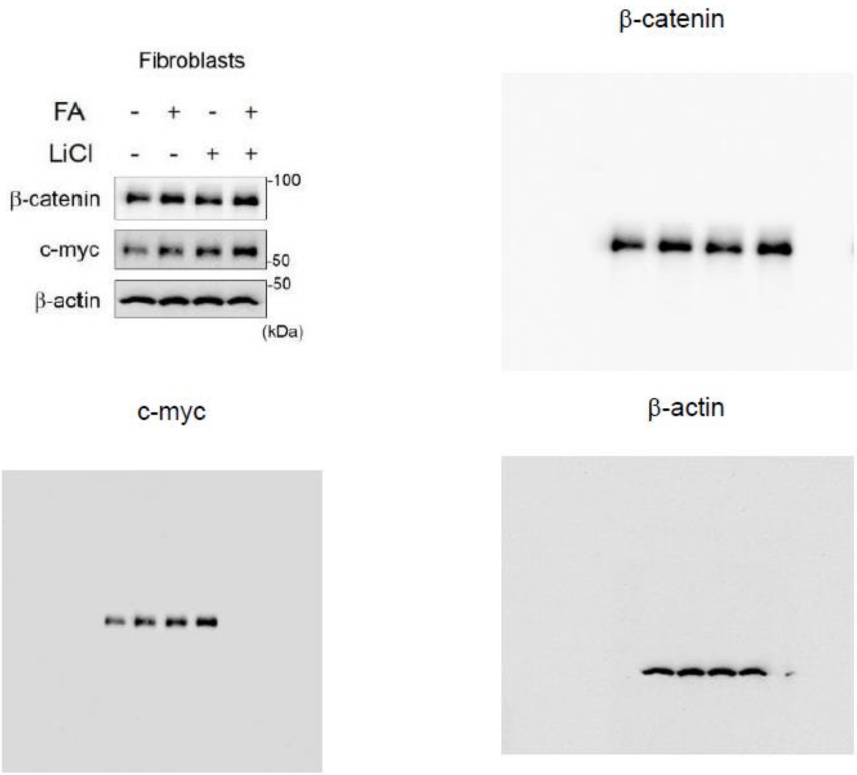

Western blotting on supplementary figure . 3

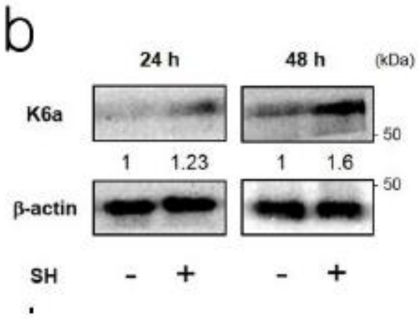

24h

K6α

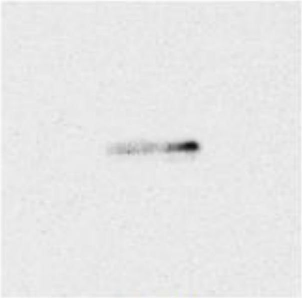

β-actin

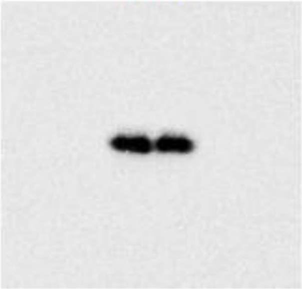

48h

K6α

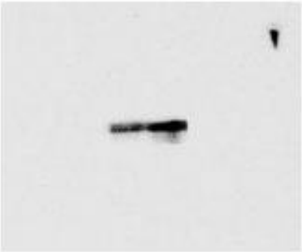

β-actin

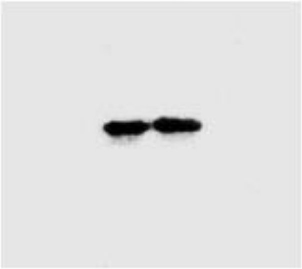

Supplement: Supplementary file 1 [file biomedicines-09-00459-s001.zip › biomedicines-1175382-supplementary.pdf]
